# Fast High-Order Consensus Time Synchronization Protocol in Industrial Wireless Sensor Networks

**DOI:** 10.3390/s26123787

**Published:** 2026-06-14

**Authors:** Xiang Yu, Zhaowei Wang, Zhongxin Zhang

**Affiliations:** School of Electrical and Information Engineering, Jiangsu University, Zhenjiang 212013, China; 2222307046@stmail.ujs.edu.cn (X.Y.); 2212407064@stmail.ujs.edu.cn (Z.Z.)

**Keywords:** time synchronization, industrial wireless sensor networks (IWSNs), fast convergence, average consensus, virtual link

## Abstract

Slow convergence remains a critical limitation hindering the practical deployment of consensus-based time synchronization protocols (CTSPs). Increasing algebraic connectivity is a key mechanism for improving the convergence speed of distributed algorithms. However, existing strategies inevitably introduce redundant packet communication, while forwarding stale timing information may degrade synchronization accuracy. To address this challenge, this paper proposes a high-order consensus time synchronization protocol (HCTSP). Unlike traditional CTSPs, HCTSP incorporates previous clock states from two-hop neighboring nodes to establish a virtual topology and further employs this information to enhance the estimation of logical clock parameters, thereby achieving fast estimation of clock parameters while effectively suppressing fluctuations in parameter estimation caused by a large initial synchronization error. Although the proposed method utilizes single-hop communication to relay information from non-adjacent nodes—an indirect transmission mechanism that inherently introduces additional communication overhead—we further develop an accumulator-based redundant information optimization scheme. Furthermore, the consensus algorithm integrates both an accumulator-based update mechanism and multi-hop historical memory, partially alleviating the impact of Gaussian communication delay jitter on clock correction. Theoretical proofs have verified the fast convergence of the proposed protocol. Extensive simulation experiments also demonstrate the superior efficiency of HCTSP in terms of convergence speed, communication overhead, and synchronization accuracy. Specifically, in random networks with 25 nodes, there is an approximately 50% reduction in single-round synchronization message length and a 66.67% decrease in total packet exchange volume compared to the virtual topology-based time synchronization protocol (VTSP). In the typical ring topology, where the convergence speed of the consensus algorithm is slow, HCTSP has a 59.33% increase in convergence speed compared to VTSP and a 75.29% increase compared to the gradient time synchronization protocol (GTSP).

## 1. Introduction

Time synchronization serves as a critical foundation for cooperative operations in distributed systems, particularly in industrial wireless sensor networks (IWSNs). The absence of precise time sequential support can significantly diminish the value of sensor-collected data and sensed information, thereby compromising the decision-making capabilities of upper-layer applications. For instance, in event-triggered control systems [1], consistent timestamps are essential to ensure accurate calculation of trigger thresholds. Similarly, in smart grid applications [2], phasor measurement units rely on high-precision time synchronization to enable synchronized sampling across the entire power distribution network.

Distributed consensus-based time synchronization protocols (CTSPs) have emerged as a prominent direction in time synchronization research due to their robust nature and inherent adaptability to the dynamic topologies characteristic of wireless sensor networks [3,4,5]. Extensive research has yielded a diverse array of CTSPs to address various performance objectives in complex network environments with disturbances, including communication delays [6,7], node mobility [8,9], security threats [10], et al. However, most CTSPs suffer from slow convergence due to their reliance on high-frequency iterative communication between adjacent nodes, especially the average-consensus-based time synchronization algorithms. Despite significant advancements in accelerating convergence speed, the multi-hop communication strategy often incurs substantial communication overhead and computational complexity [11]. This issue is particularly pronounced in large-scale, dense, and resource-constrained IWSNs, where communication delays and path-dependent accumulation of estimation errors are inevitable under multi-hop communication protocols [12]. Hence, this paper focuses on optimizing time synchronization convergence speed while balancing critical performance factors, including communication overhead and delay tolerance.

### 1.1. Related Work and Motivation

Over the past decades, research on time synchronization protocols has made substantial progress, with architectures broadly categorized into centralized and distributed paradigms. Early centralized architectures predominantly rely on reference nodes and specific topology management schemes, which result in poor adaptability to dynamic network topology. Hence, recent advancements have investigated distributed average consensus protocols, such as the gradient time synchronization protocol (GTSP) [4] and the average timesync (ATS) [5]. While average consensus protocols entirely relying on neighbor information iteration demonstrate strong robustness and scalability, they often fail to satisfy real-time convergence demands. To realize finite-time convergence, He et al. proposed maximum-value time synchronization (MTS) [3], which eliminates redundant iterations by anchoring convergence to the network’s fastest clock. Unfortunately, this protocol relies on comparing the relative clock parameters between two nodes for local clock adjustments, making it highly sensitive to delay jitter.

Currently, some studies have increasingly focused on optimizing the convergence speed of CTSPs, which we systematically classify into three distinct categories.

#### 1.1.1. Cluster-Based Optimization Scheme

Recent research in cluster-based consensus time synchronization has demonstrated significant improvements in convergence efficiency. Wu et al. proposed a hierarchical network architecture to shorten the communication hops and enable parallel distributed processing [13]. In addition, Wang et al. proposed a three-step optimization scheme [14] to further reduce the synchronization overhead, which introduces a cluster-head logical skew threshold to control the cluster member synchronization and adopts a one-way communication method to reduce communication at the algorithm level. Liu et al. constructed a nearest K-neighboring (NKN) topology via K-means and proposed the TS-NKN algorithm [15] to balance convergence speed, accuracy, and energy consumption. While these cluster approaches enhance synchronization convergence efficiency and reduce communication consumption through structured consensus design, they face inherent limitations in dynamic network environments. As sensor nodes continuously consume energy, the communication radius of cluster head nodes gradually shrinks, causing a reduction in the number of overlapping clusters. This degradation may lead to slower convergence speed and potential block-level independent synchronization, ultimately compromising global synchronization stability.

#### 1.1.2. Hybrid Scheme

Some studies have also attempted to integrate the advantages of different algorithms to accelerate the convergence of consensus protocols. Zhao et al. proposed a max–average consensus protocol [16], which employs maximum consensus for logical skew compensation to eliminate redundant calculations and average consensus for logical offset compensation to effectively suppress synchronization error fluctuations. The hybrid time synchronization protocol (HTSP) [17] integrates reference-based and consensus-based models through dynamic mode switching. Pei et al. proposed a communication-aware distributed training (CADT) framework [18], which utilizes real-time CSI for dynamic gradient aggregation to enhance convergence speed and accuracy in wireless environments. However, these hybrid approaches inevitably compromise the robustness and scalability inherent in fully distributed protocols. This performance trade-off is particularly evident in large-scale dynamic networks, especially in terms of adaptability to topological changes (e.g., node addition and node mobility), where performance degradation may limit the practical applicability of hybrid-architecture-based protocols.

#### 1.1.3. Multi-Hop Virtual Communication

Reducing network diameter and enhancing algebraic connectivity [19] constitute fundamental prerequisites for accelerating the convergence of consensus algorithms. Based on this theoretical premise, Panigrahi et al. proposed the selective average time synchronization (SATS) protocol [20], which employs a dynamic programming mechanism to identify the optimal multi-hop path with minimal synchronization error under constrained conditions. To increase algebraic connectivity, Shi et al. developed the multi-hop average consensus time synchronization (MACTS) protocol [21] based on the multi-hop relay concept, where nodes continuously forward multi-hop information during non-broadcast cycles to establish virtual communication links. While forwarding-based multi-hop virtual communication techniques effectively improve synchronization convergence speed, they inevitably incur a significant increase in communication overhead. More critically, in large-scale practical IWSNs, high-frequency updates may induce node contention, and the forwarding process also introduces uncertain transmission delays, directly resulting in synchronization errors between multi-hop nodes and local nodes. Furthermore, it is challenging to predefine an appropriate error threshold in real-world deployments. For distributed algorithms that rely solely on neighbor information to maintain system states, the lack of global topology knowledge makes it difficult to determine a proper threshold. In addition, a single fixed threshold cannot effectively adapt to dynamic network environments, such as random node joining and leaving.

To address the limitations of [3,21], Wang et al. proposed the virtual maximum-value time synchronization (VMTS) protocol [22], which is based on multi-hop links. In this protocol, an estimator based on the moving-average principle is designed to minimize the impact of normally distributed random delays on the maximum consensus algorithm, thereby improving synchronization accuracy. Shi et al. subsequently introduced a parameter-sharing strategy [23], which uses single-hop communication instead of forwarding every received message for multi-hop communication. By sharing relative clock offset estimations with neighboring nodes, virtual links are established with multi-hop nodes, thereby simplifying algorithm complexity and reducing information exchange. Duan et al. [24] dynamically adjusted the communication hop count in real time according to synchronization error states and network topology variations through online estimation of the convergence probability and its variation rate of synchronization errors, enabling a more practical and accurate design of multi-hop consensus algorithms. Unlike ATS-based asynchronous virtual communication [21,23], Phan et al.’s synchronous virtual topology-based time synchronization protocol (VTSP) [25] updates clock parameters in a manner similar to GTSP. However, VTSP only incorporates neighboring information when broadcasting local timing information during each synchronization period, thereby reducing the communication cost associated with the multi-hop forwarding strategy. To further optimize communication efficiency, Wang et al. enhanced the virtual multi-hop synchronization framework by incorporating an event-triggered mechanism [26]. This method significantly reduces redundant broadcasts compared with periodic synchronization approaches [21,23,25].

The aforementioned multi-hop virtual communication synchronization algorithms primarily accelerate the convergence of consensus algorithms by utilizing current states or forwarded neighboring information to improve the algebraic connectivity of the network. However, these methods have not yet fully exploited the potential of historical memory information for accelerating consensus convergence [27,28]. The work in [29] has shown that, in most cases, multi-step consensus algorithms incorporating local historical memory can achieve superior convergence performance compared with conventional gradient descent methods, and their effectiveness has been validated in distributed average consensus engineering applications. Furthermore, [30] analyzed the theoretical bounds of the fastest achievable convergence rate under different tap conditions (i.e., memory orders), as well as the corresponding optimal parameter design problem.

Although expanding the communication range and increasing node degrees are fundamental methods for enhancing a graph’s algebraic connectivity, they inevitably lead to increased power consumption. This trade-off makes such approaches unsuitable for low-power IWSNs. The aforementioned multi-hop virtual communication methods have demonstrated notable improvements in convergence speed and communication efficiency. However, compared with conventional single-hop consensus synchronization protocols [3,4,5], these methods may potentially compromise synchronization accuracy and convergence performance due to the introduction of uncertain relay delays [21] and non-real-time multi-hop neighboring timing information [23,25,26]. Moreover, in dense network deployments, the single-hop communication mechanism carrying multi-hop timing information still incurs substantial communication overhead [25]. Consequently, this motivates us to seek a time synchronization protocol for IWSNs that achieves fast convergence, low communication overhead, and delay tolerance.

### 1.2. Contribution

Existing studies have demonstrated that incorporating historical state information can enhance the convergence speed of distributed systems [27,28,29,30,31]. Motivated by this idea, the historical information of two-hop neighboring nodes is incorporated into the virtual-link construction mechanism to alleviate the update-direction bias induced by uncertain two-hop terms. Based on this core principle, we propose a novel high-order consensus time synchronization protocol (HCTSP) to overcome the challenge of slow convergence in current CTSPs while maintaining low communication overhead and delay tolerance. The main contributions are as follows.

1. The proposed joint skew-offset estimation framework utilizes both the current clock states of one-hop neighboring nodes and the historical clock states of two-hop neighboring nodes to estimate relative clock parameters. Rigorous theoretical proofs of the rapid convergence of the proposed method are provided through comparison with traditional multi-hop synchronization protocols.
2. The design of HCTSP is based on a simple single-hop communication model, which greatly reduces communication volume compared with multi-hop relay communication. Moreover, to reduce the additional packet overhead associated with multi-hop neighboring information, an accumulator-based redundant information optimization scheme is proposed.
3. By integrating historical information from multi-hop neighboring nodes, the proposed method achieves superior link-state observability, leading to improved synchronization accuracy in virtual multi-hop networks. Extensive simulation results demonstrate that HCTSP can accelerate convergence with low overhead under various network topologies while effectively improving synchronization accuracy. Notably, the method maintains compatibility with both GTSP and ATS protocols.

The remainder of this article is organized as follows. Section 2 introduces the system model and analyzes average consensus problem. Section 3 proposes the fast high-order consensus protocol. Section 4 presents the implementation of HCTSP. Section 5 validates and discusses the simulation results of the proposed HCTSP. Section 7 concludes the main work.

## 2. System Model and Problem Formulation

An IWSN can be modeled as an undirected graph G=(V,E), where V=1,2,3,⋯,n denotes the set of nodes and *E* represents the set of available communication links between nodes, also known as the edge set. (i,j)∈E indicates that nodes *i* and *j* can maintain bidirectional information exchange. The set $N_{i}=j|(i,j)\in E,\;i\neq j$, ∀i∈V, corresponds to the neighbors of node *i*. Obviously, $\left|N_{i}\right|$ denotes the number of neighbors and is also defined as the degree of node *i* in graph theory. Consequently, the degree matrix *D* of graph *G* is a diagonal matrix with $D_{ii}=\deg\left(i\right)$. Let *A* denote the adjacency matrix. If (i,j)∈E, the element $a_{ij}$ in *A* is 1; otherwise, it is 0.

The Laplacian matrix *L* is calculated as L=D−A. By definition, *L* is positive semidefinite, and its eigenvalues can be ordered as $0=\lambda_{1}\leq \lambda_{2}\leq \cdots \leq \lambda_{n}$. Here, the second-smallest eigenvalue $\lambda_{2}$, also known as the algebraic connectivity, is a necessary and sufficient condition for the connectedness of an undirected graph *G*.

### 2.1. Clock Model

Usually, each node in IWSN maintains a local hardware clock $H_{i}\left(t\right)$, which follows an affine model [3,4,5],

(1) $$H_{i}\left(t\right)=\alpha_{i}t+\beta_{i}$$

where *i* denotes the node ID and *t* represents the absolute time. $\alpha_{i}$ is defined as the clock skew, which describes the ratio of the actual frequency to the nominal frequency. The clock offset $\beta_{i}$ represents the initial phase difference at startup. Consistent with prior work [4], it is assumed that the clock skew is bounded, i.e., $1-\sigma <\alpha_{i}<1+\sigma$, where σ∈[30,100] ppm. For practical applications, environmental variations and oscillator aging change slowly; therefore, it can be reasonably assumed that the skew $\alpha_{i}$ remains constant within a short synchronization interval. Since local nodes lack access to an absolute time reference, direct computation and adjustment of the hardware clock $H_{i}\left(t\right)$ are not feasible. To address this issue, the concept of a logical clock is introduced to represent the synchronized clock among nodes, i.e.,

(2) $$L_{i}\left(t\right)={\hat{\alpha }}_{i}\left(t\right)H_{i}\left(t\right)+{\hat{\beta }}_{i}\left(t\right)={\hat{\alpha }}_{i}\left(t\right)\alpha_{i}t+{\hat{\alpha }}_{i}\left(t\right)\beta_{i}+{\hat{\beta }}_{i}\left(t\right)$$

where ${\hat{\alpha }}_{i}\left(t\right)$ and ${\hat{\beta }}_{i}\left(t\right)$ denote the compensation parameters for logical clock skew and offset, respectively. $s_{i}={\hat{\alpha }}_{i}\left(t\right)\alpha_{i}$ and $o_{i}={\hat{\alpha }}_{i}\left(t\right)\beta_{i}+{\hat{\beta }}_{i}\left(t\right)$ are referred to as the logical clock skew and offset of node *i*. When the logical clocks of all nodes converge to a common value, the network achieves global synchronization, which means that

(3) $$\lim_{t\to \infty }\left|L_{j}\left(t\right)-L_{i}\left(t\right)\right|=0,\forall i,j\in V$$

In distributed CTSPs, where no fixed global reference node exists, the relative clock skew $\alpha_{ij}$ and offset $\beta_{ij}$ serve as two fundamental parameters for characterizing differences between two clocks. Specifically, $\alpha_{ij}=\alpha_{j}/\alpha_{i}$ reflects the frequency ratio between two oscillators and is estimated using two pairs of hardware timestamps, i.e., $\alpha_{ij}\left(t\right)=\left(H_{j}\left(t\right)-H_{j}\left(t-1\right)\right)/\left(H_{i}\left(t\right)-H_{i}\left(t-1\right)\right)$. $\beta *ij\left(t\right)=L*j\left(t\right)-L_{i}\left(t\right)$ is used to describe the instantaneous logical clock difference between nodes *i* and *j*.

### 2.2. Average-Based Consensus Time Synchronization Problem

It can be observed that the core requirement of (3) is to achieve dual convergence of both logical clock skew and offset states, i.e., $\lim_{t\to \infty }\left|s_{j}-s_{i}\right|=0$, $\lim_{t\to \infty }\left|o_{j}-o_{i}\right|=0$, ∀i,j∈V. Usually, CTSPs employ a linear consensus model to describe the synchronization process [5,6]. The dynamic equation is given as follows [32]:

(4) $$x_{i}\left(k+1\right)=x_{i}\left(k\right)+\sum_{j\in N_{i}}a_{ij}\left(x_{j}\left(k\right)-x_{i}\left(k\right)\right)$$

where $x_{i}$ represents the clock state of node *i*. If $x_{i}=x_{j}$, ∀i,j∈V, this indicates that the states of all nodes are consistent. Let $x_{i}\left(0\right)$ denote the initial state, and let *x* denote the asymptotic convergence state of the system. As established in [19,32], the final convergence result of (4) is equal to the average of the initial clock states of all nodes, that is,

(5) $$\lim_{t\to \infty }x=\frac{1}{n}\sum_{i=1}^{n}x_{i}\left(0\right)$$

The convergence speed of average-based consensus theory is fundamentally governed by the algebraic connectivity $\lambda_{2}$ of the network Laplacian graph [32], exhibiting a direct positive correlation, where faster convergence is achieved with a larger value of $\lambda_{2}$. Although expanding the communication radius of nodes, increasing network density [11], and constructing virtual links [21,23,25,26] to create richer interconnections can effectively increase algebraic connectivity, these schemes inevitably introduce practical limitations, including increased hardware costs, packet collisions, network congestion, power consumption, and delay jitter. To overcome the above challenges, this paper designs a high-order consensus protocol.

## 3. Fast High-Order Consensus Protocol

The proposed high-order consensus protocol is an innovative extension of virtual communication mechanism, aimed at utilizing the historical state information of 2-hop neighbors to achieve fast convergence. To reduce the unnecessary overhead of relay communication, the proposed protocol strategically retains a single-hop communication architecture. The high-order consensus dynamic equation can be formulated as

(6) $$\begin{array}{cc} x_{i}\left(k+1\right) & =x_{i}\left(k\right)+\varepsilon \sum_{j\in N_{i}}a_{ij}\{(x_{j}\left(k\right)-x_{i}\left(k\right)\\ \; & +\sum_{l\in N_{j}}a_{jl}\left(x_{l}\left(k-1\right)-x_{i}\left(k\right)\right)+\cdots \\ \; & +\sum_{l\in N_{j}}a_{jl}\left(x_{l}\left(k-m\right)-x_{i}\left(k\right)\right)\} \end{array}$$

where $x_{i}\left(k\right)$ denotes the clock state of node *i* at time *k*. ε is a weight parameter. Node *j* is the 1-hop neighbor of node *i*, while node *l* is the 2-hop neighbor. At *k*-th broadcast times, node *j* will send its current clock status $x_{j}\left(k\right)$ to node *i* along with node *l*’s historical state $x_{l}\left(k-m\right)$. It should be noted that rather than being directly appended to node *j*’s packet, multiple $x_{l}\left(k-m\right)$ are utilized to construct a new smaller parameter, thereby reducing additional packet size. The implementation details will be thoroughly described in the design of HCTSP in Section 4.

### 3.1. Convergence Analysis

We begin the convergence analysis of high-order consensus protocol by reformulating (6) in vector form

(7) $$\begin{array}{cc} x(k+1) & =\left(I_{n}-\varepsilon L-m\varepsilon D_{2}\right)x\left(k\right)+\varepsilon A_{2}\sum_{t=1}^{m}x\left(k-t\right) \end{array}$$

where $x\left(k\right)={\left[x_{1}\left(k\right),x_{2}\left(k\right),\cdots ,x_{n}\left(k\right)\right]}^{T}$, $A_{2}$ is the adjacency matrix of the 2-hop topology of G=(V,E). Here, $D_{2}=\mathrm{diag}\left(\sum_{j\in N_{i}}a_{ij}\sum_{l\in N_{j}}a_{jl}\right)$, i≠l, ∀i,j,l∈V.

Thus (7) can be rewritten as

(8) X(k+1)=CX(k)

where $X\left(k+1\right)={\left[x(k+1),\cdots x(k-m+1)\right]}^{T}$, and

(9) $$C=\left[\begin{array}{ccccc} I_{n}-\varepsilon L-m\varepsilon D_{2} & \varepsilon A_{2} & \cdots & \cdots & \varepsilon A_{2}\\ I_{n} & 0 & \cdots & \cdots & 0\\ 0 & I_{n} & \cdots & \cdots & 0\\ \vdots & \vdots & \ddots & \vdots & \vdots \\ 0 & 0 & \cdots & I_{n} & 0 \end{array}\right]$$

Let

(10) $$J=\left[\begin{array}{cccc} K & 0 & \cdots & 0\\ K & 0 & \cdots & 0\\ \vdots & \vdots & \; & \vdots \\ K & 0 & \cdots & 0 \end{array}\right]$$

where $K=\frac{1}{n}11^{T},1={\left[1,1,\cdots ,1\right]}^{T}$ is an *n*-dimensional vector.

**Lemma** **1.** *C shares identical eigenvalues with matrix C−J, except that λ(C)=1 is replaced by λ(C−J)=0.*

**Proof.**  Construct a matrix $\tilde{C}=C-1v^{T}$, where $v\in R^{n}$ is an arbitrarily given *n* dimensional vector. For any invertible matrix C, according to the determinant lemma [33],

(11) $$\begin{array}{c} \det\left(\tilde{C}-\lambda_{\tilde{c}}I_{n}\right)=\det\left(C-1v^{T}-\lambda_{\tilde{c}}I_{n}\right) \end{array}$$

Next, we discuss the invertibility of matrix $C-\lambda_{\tilde{c}}I_{n}$. After performing elementary row operations on $C-\lambda_{\tilde{c}}I_{n}$, it strictly satisfies the upper triangular matrix form. Therefore, it is easy to prove that when $\lambda_{\tilde{c}}\neq 0$, $\lambda_{\tilde{c}}\neq 1$ and $\lambda_{\tilde{c}}\neq 1-\varepsilon \lambda_{k}$, Matrix $C-\lambda_{\tilde{c}}I_{n}$ is invertible.

Thus, (11) can be further applied

(12) $$\begin{array}{c} \det\left(\tilde{C}-\lambda_{\tilde{c}}I_{n}\right)=\det\left(C-\lambda_{\tilde{c}}I_{n}\right)\cdot \left(1-v^{T}{\left(C-\lambda_{\tilde{c}}I_{n}\right)}^{-1}1\right) \end{array}$$

The characteristic equation is

(13) $$\det\left(C-\lambda_{\tilde{c}}I_{n}\right)\cdot \left(1-v^{T}{\left(C-\lambda_{\tilde{c}}I_{n}\right)}^{-1}I_{n}\right)=0$$

From (13), there is (i) if $\lambda_{c}\neq 1$, $\det(C-\lambda_{\tilde{c}}I_{n})=0$; that is, $\lambda_{\tilde{c}}$ is an eigenvalue of C; (ii) if $\lambda_{c}=1$, $1-v^{T}{\left(C-\lambda_{\tilde{c}}I_{n}\right)}^{-1}I_{n}=0$; that is, $\lambda_{\tilde{c}}=0$ is replaced by $\lambda_{c}=1$. Therefore, the proof is complete.    □

**Theorem** **1.** *For an undirected graph G that is strongly connected, the higher-order consensus protocol ensures convergence to the average of the initial stages if the spectral radius condition ρ(C−J)<1 holds under the given initial conditions x(0)=x(−1)=⋯x(−m+1)* [31].

**Proof.**  First, We have a region *S* that satisfies ρ(C−J)<1; that is to say,

(14) S={ε∣ρ(C−J)<1}

if and only if

(15) $$0<\varepsilon <\frac{2}{\lambda_{1}\left(L+mD_{2}+\left(m-1\right)A_{2}\right)}$$

where $\lambda_{i}\left(\cdot \right)$ denotes the *i*th largest eigenvalue of a symmetric matrix. The proof regarding the spectral radius in Theorem 1 is similar to [34]. Here, we present a sketch proof.    □

In the orthogonal complement subspace, K1=1, and for u⊥1, Ku=0. So the matrix C−J reduces to a block companion matrix, and its eigenvalues satisfy $\det(\mu_{i}I_{n}-W+\varepsilon A_{2}\left(\frac{1}{\mu_{i}}+\cdots +\frac{1}{\mu_{i}^{m-1}}\right))=0$, where $W=I_{n}-\varepsilon L-m\varepsilon D_{2}$. Since C depends on ε and $\mu_{i}=1$ at ε=0, a perturbation expansion gives $\mu_{i}=1-\varepsilon \lambda_{i}+O\left(\varepsilon^{2}\right)$, where $\lambda_{i}$ are eigenvalues of a reference matrix (e.g., $L+mD_{2}+\left(m-1\right)A_{2}$). These $\lambda_{i}$ appear in the upper bound condition later.

For the dominant eigenvalue, the characteristic equation can be approximated as $\det\left(\left(1-\varepsilon \lambda_{i}\right)I_{n}\right)-W+\left(m-1\right)\varepsilon A_{2})=0$. The requirement is $|\mu_{i}|\;<\;1$, i.e., $|1-\varepsilon \lambda_{i}|\;<\;1$. Moreover, given the positive semi-definiteness of L and that $D_{2}$ is a non-negative matrix, $0<\varepsilon <\frac{2}{\lambda_{1}\left(L+mD_{2}+\left(m-1\right)A_{2}\right)}$.

For non-dominant eigenvalues ($\mu_{i}\ll 1$), consider the exact characteristic equation of C restricted to the subspace orthogonal to 1:

(16) $$\det(\mu^{m}I_{n}-\mu^{m-1}\left(I_{n}-\varepsilon L-m\varepsilon D_{2}\right)-\varepsilon A_{2}\left(1+\mu +\cdots +\mu^{m-1}\right))=0.$$

Let v be a unit eigenvector with v⊥1 corresponding to μ. Rearranging the eigenvalue equation gives

(17) $$\mu =1-\varepsilon v^{T}Lv-m\varepsilon v^{T}D_{2}v+\varepsilon v^{T}A_{2}v\cdot \frac{1-\mu^{m}}{\mu^{m-1}\left(1-\mu \right)}.$$

We now show that under condition (15), every such μ satisfies |μ|<1. Assume, to the contrary, that |μ|≥1 and μ≠1. Because L and $D_{2}$ are positive semi-definite, we have $v^{T}Lv\geq \lambda_{2}>0$ (where $\lambda_{2}$ is the algebraic connectivity) and $v^{T}D_{2}v\geq 0$. Moreover, for the symmetric matrix $A_{2}$, the Rayleigh quotient satisfies $|v^{T}A_{2}v|\leq \rho \left(A_{2}\right)$ (spectral radius).

(18) $$\left|\frac{1-\mu^{m}}{\mu^{m-1}\left(1-\mu \right)}\right|=\left|\frac{1+\mu +\cdots +\mu^{m-1}}{\mu^{m-1}}\right|\leq \frac{1+\left|\mu \right|+\cdots +{\left|\mu \right|}^{m-1}}{{\left|\mu \right|}^{m-1}}\leq m.$$

Taking absolute values in the previous equation and using the triangle inequality yields

(19) $$\left|\mu \right|\leq 1-\varepsilon v^{T}Lv-m\varepsilon v^{T}D_{2}v+m\varepsilon \left|v^{T}A_{2}v\right|.$$

Denote $a=v^{T}Lv$, $b=v^{T}D_{2}v$, $c=|v^{T}A_{2}v|$. Then the inequality becomes |μ|≤1−εa−mεb+mεc. Using the Rayleigh quotient bounds and condition (15), we have $\varepsilon a\geq \varepsilon \lambda_{2}$ and $\varepsilon c\leq \varepsilon \rho (A_{2})$. Since ε is bounded by $\frac{2}{\lambda_{1}\left(L+mD_{2}+\left(m-1\right)A_{2}\right)}$, a direct calculation (see, e.g., the Gershgorin circle argument in [34]) shows that 1−εa−mεb+mεc<1. Hence |μ|<1, contradicting the assumption |μ|≥1. Therefore all non-dominant eigenvalues satisfy $|\mu_{i}|<1$ and decay exponentially.

In the all-ones space, the action of matrix C−J is dominated by the low-dimensional subsystem. Since $D_{2}1=A_{2}1$, when ε is sufficiently small, the perturbation term $m\varepsilon D_{2}1$ is not enough to make the modulus exceed 1, so the modulus of this subspace is also less than 1. In summary, when $0<\varepsilon <\frac{2}{\lambda_{1}\left(L+mD_{2}+\left(m-1\right)A_{2}\right)}$, the condition ρ(C−J)<1 is satisfied.

Next, we continue the proof of Theorem 1 under the constraint condition. For the undirected communication topology *G*, both L and $L_{2}$ are symmetric matrices with zero row and column sums. Hence, C has row sums equal to 1 and must possess an eigenvalue of 1. By Lemma 1, matrix C, except for the eigenvalue 1, has the same eigenvalues as matrix C−J. When C−J satisfies the spectral radius condition ρ(C−J)<1, C consequently has a unique eigenvalue of 1 and the remaining eigenvalues satisfy |λ(C)|<1.

Furthermore, for the eigenvalue 1 of C, there exists a left eigenvector $\omega_{l}$ and a right eigenvector $\omega_{r}$, respectively. Let matrix U be the Jordan canonical form of matrix C and S be its corresponding eigenvector matrix, such that $C=SUS^{-1}$; then, $C^{k}=SU^{k}S^{-1}$. Hence,

(20) $$\begin{array}{cc} \lim_{k\to \infty }C^{k} & =\lim_{k\to \infty }{\left[\begin{array}{ccccc} I_{n}-\varepsilon L-m\varepsilon D_{2} & \varepsilon A_{2} & \cdots & \cdots & \varepsilon A_{2}\\ I_{n} & 0 & \cdots & \cdots & 0\\ 0 & I_{n} & \cdots & \cdots & 0\\ \vdots & \vdots & \ddots & \vdots & \vdots \\ 0 & 0 & \cdots & I_{n} & 0 \end{array}\right]}^{k}=\omega_{l}\omega_{r}^{T} \end{array}$$

Define $\omega_{l}={\left(1^{T},0^{T},\cdots ,0^{T}\right)}^{T}$, $\omega_{r}={\left(1^{T},1^{T},\cdots ,1^{T}\right)}^{T}$. According to the above equation, there is

(21) $$\lim_{k\to \infty }\left[\begin{array}{c} X(k+1)\\ X(k)\\ \vdots \\ X(k-m+2) \end{array}\right]=\omega_{r}\omega_{l}^{T}\left[\begin{array}{c} X(0)\\ X(-1)\\ \vdots \\ X(-m+1) \end{array}\right]$$

i.e., $\lim_{k\to \infty }X\left(k+1\right)=\lim_{k\to \infty }\mathrm{CX}\left(k\right)=\omega_{r}\omega_{I}^{T}X\left(0\right)$. So we can conclude that $\lim_{k\to \infty }x\left(k+1\right)=\frac{1}{n}1^{T}\sum_{i=1}^{n}x_{i}\left(0\right)$. The proof of Theorem 1 is complete.

### 3.2. Convergence Speed Analysis

The convergence speed of distributed consensus algorithms can be intuitively compared through the second smallest eigenvalue of the Laplacian matrix [35]. However, given the computational complexity of exact eigenvalue calculation, a simplified method is employed to roughly compare the convergence speed between high-order consensus algorithms and multi-hop consensus algorithms [11] under identical hop-count constraints.

Considering the 2-hop algorithm as a representative case, we first rewrite (7) in high-order consistent vector form as

(22) $$\begin{array}{cc} x(k+1)= & \left(I_{n}-\varepsilon L-m\varepsilon D_{2}+m\varepsilon A_{2}\right)x\left(k\right)+\varepsilon A_{2}\left[x\left(k-1\right)-x\left(k\right)\right]\\ \; & +\cdots +\varepsilon A_{2}\left[x\left(k-m\right)-x\left(k\right)\right] \end{array}$$

As rigorously proved in the previous section, if the high-order consensus algorithm satisfies the convergence condition, i.e.,

(23) $$\lim_{k\to \infty }x\left(k+1\right)=\lim_{k\to \infty }x\left(k\right)=\cdots \lim_{k\to \infty }x\left(k-m\right)$$

After a finite number of iterations, ${∥x(k)-x(k-1)∥}_{2}\to 0$,⋯, ${∥x(k)-x(k-m+1)∥}_{2}\to 0$. Thus, we can get the following approximation

(24) $$\begin{array}{cc} x(k+1) & =\left(I_{n}-\varepsilon L-mD_{2}+m\varepsilon A_{2}\right)x\left(k\right)=\left(I_{n}-\varepsilon L-m\varepsilon L_{2}\right)x\left(k\right) \end{array}$$

For a traditional first-order 2-hop relay forwarding algorithm, it can also be approximated as

(25) $$x\left(k+1\right)\approx \left(I_{n}-\varepsilon L-\varepsilon L_{2}\right)x\left(k\right)$$

Therefore, the convergence speed comparison between the high-order consensus algorithm and the traditional multi-hop algorithm can be achieved by analyzing the second smallest eigenvalue of the matrices $\left(I_{n}-\varepsilon L-m\varepsilon L_{2}\right)$ and $\left(I_{n}-\varepsilon L-\varepsilon L_{2}\right)$. Due to the symmetric positive semi-definiteness of the Laplacian matrices L, $L_{2}$, it is obvious that when m>1, the high-order consensus algorithm converges faster.

## 4. Fast Time Synchronization Protocol: HCTSP

**Figure 1:**
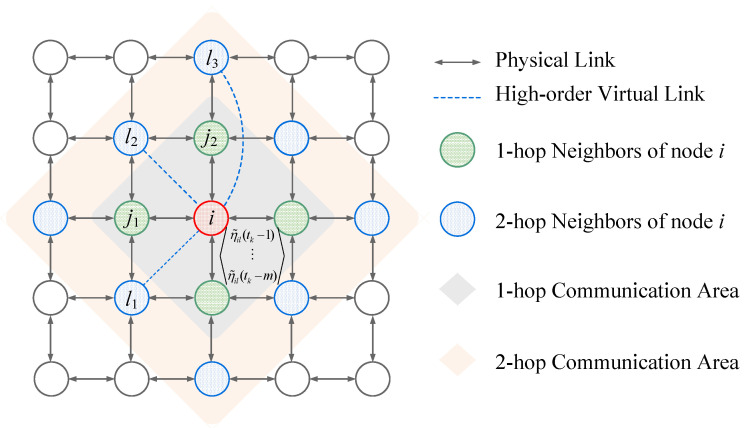
2-hop HCTSP indication. Illustrates how local node *i* establishes a virtual link with multi-hop node *l* by receiving synchronization packet data from neighbor node *j*.

The core idea of HCTSP is to exchange multi-hop clock information within a single-hop communication model to establish virtual links between local nodes and their multi-hop neighboring nodes. As illustrated in Figure 1, nodes *j* and *l* are the one-hop and two-hop neighbors of node *i*, respectively. In each broadcast round, node *j* transmits its local clock state together with information from its single-hop neighbor *l*. Meanwhile, node *i* utilizes both one-hop and two-hop clock information to update its local clock. As a result, virtual communication links can be established between node *i* and its nonadjacent node *l*.

**Figure 2:**
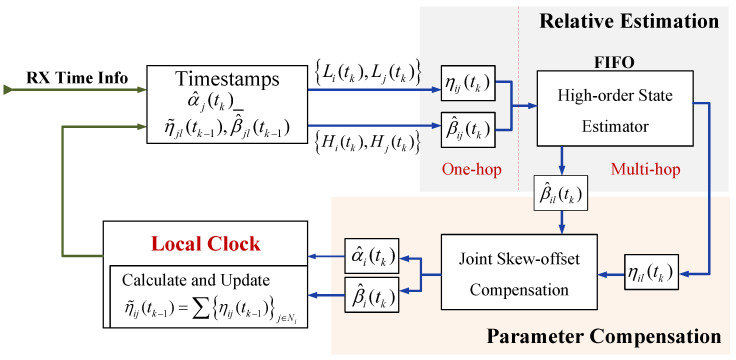
Implementation of HCTSP. RX time information from neighboring node *j* is utilized for one-hop estimation and multi-hop synchronization.

Figure 2 presents the detailed workflow of HCTSP. The proposed HCTSP primarily consists of two key phases: relative clock parameter estimation and logical clock compensation value calculation.

### 4.1. Relative Clock Parameter Estimation

Clock skew and offset are two fundamental parameters governing logical clock behavior in distributed synchronization protocols. Within these protocols, nodes iteratively process timestamps from neighboring nodes to calculate and update their local clock states. During the update process, accurately estimating relative clock parameters between nodes is crucial, as this estimation forms the foundation for achieving high-precision time synchronization. In HCTSP, this estimation process occurs at two levels: between directly adjacent nodes and among multi-hop connected nodes. Furthermore, to mitigate the impact of uncertain delays caused by timing message delivery, a media access control (MAC) layer-based physical timestamp is adopted [3].

#### 4.1.1. Relative Parameter Estimation Between Adjacent Nodes

Upon receiving timestamps from adjacent node *j* at $t_{k}$, node *i* records the arrival time and estimates the relative clock skew $\alpha_{ij}$ and offset β∗ij by employing two successive timestamp pairs, as in regular CTSPs. To clearly express the clock adjustment process, we define the following notation $\eta *ij(t_{k})$ as the skew compensation of node *i* calculated from the timing information of neighboring node *j*.

(26) $$\eta_{ij}\left(t_{k}\right)=\alpha_{ij}\left(t_{k}\right){\hat{\alpha }}_{j}\left(t_{k}\right)$$

#### 4.1.2. Relative Parameter Estimation Among Non-Adjacent Nodes

Since hardware timestamp information cannot be directly acquired from non-adjacent nodes and to minimize communication overhead from relay forwarding, HCTSP avoids direct hardware timestamp transmission in virtual communication. However, the relative clock skew between node *i* and its non-adjacent node *l* can be derived indirectly through the expression $\alpha_{il}\left(t_{k}\right)=\alpha_{ij}\left(t_{k}\right)\alpha_{jl}\left(t_{k}\right)$. Hence, node *i*’s skew compensation calculated from node *l*’s timing information satisfies

(27) $$\eta_{il}\left(t_{k}\right)=\alpha_{ij}\left(t_{k}\right)\alpha_{jl}\left(t_{k}\right){\hat{\alpha }}_{l}\left(t_{k}\right)=\alpha_{ij}\left(t_{k}\right)\eta_{jl}\left(t_{k}\right)$$

Here, we refer to $\eta_{il}\left(t_{k}\right)$ as the first-order skew compensation.

The convergence analysis in Section 3.1 has demonstrated that incorporating multi-hop node historical information significantly accelerates the convergence speed of distributed consensus algorithms. Therefore, the real-time cumulative high-order skew compensation is

(28) $$\begin{array}{cc} {\tilde{\eta }}_{il}\left(t_{k}\right) & =\alpha_{ij}\left(t_{k}\right)\eta_{jl}\left(t_{k-1}\right)+\alpha_{ij}\left(t_{k}\right)\eta_{jl}\left(t_{k-2}\right)\\ \; & \;\;+\cdots +\alpha_{ij}\left(t_{k}\right)\eta_{jl}\left(t_{k-m}\right)\\ \; & =\alpha_{ij}\left(t_{k}\right)\sum_{n=1}^{m}\eta_{jl}\left(t_{k-n}\right) \end{array}$$

While the relative logical clock offset between node *i* and its non-adjacent node *l* can be simply estimated as

(29) $$\beta_{il}\left(t_{k}\right)=\beta_{ij}\left(t_{k}\right)+{\bar{\beta }}_{jl}\left(t_{k}\right)$$

where ${\bar{\beta }}_{jl}\left(t_{k}\right)=\frac{\sum_{l\in N_{j}}\beta_{jl}\left(t_{k-1}\right)}{\left|N_{j}\right|+1}$, i.e., node *j* sends a calculated average of ${\left\{\beta_{jl(t_{k-1})}\right\}}_{l\in N_{j}}$ to node *i*.

### 4.2. Logical Clock Compensation Values Calculation

To accelerate convergence, we design a joint skew-offset compensation calculation for node adjustment based on the fast high-order consensus protocol proposed in Section 3. The calculation process is an extension of GTSP [4].

First, we estimate the logical skew compensation parameter through high-order consensus principle; that is

(30) $${\hat{\alpha }}_{i}\left(t_{k}^{+}\right)=\frac{\sum_{j\in N_{i}}\eta_{ij}\left(t_{k}\right)+\sum_{l\in N_{i}^{\prime }}{\tilde{\eta }}_{il}\left(t_{k}\right)+{\hat{\alpha }}_{i}\left(t_{k}\right)}{|N_{i}\left|+m\right|N_{i}^{\prime }|+1}$$

where $t_{k}^{+}$ is the updated instantaneous moment and $|N_{i}^{\prime }|$ represents the number of node *i*’s 2-hop neighbors.

To prove that logical clock skew compensation (30) satisfies the high-order consensus theory, we further conduct the following verification process. It should be emphasized that the logical clock skew compensation calculation serves to synchronize the logical clock skew, i.e., $s_{i}=s_{j}$. Therefore, by multiplying both sides of (30) by $\alpha_{i}\left(t_{k}\right)$, we have

(31) $$\begin{array}{cc} {\hat{\alpha }}_{i}\left(t_{k}^{+}\right)\alpha_{i}\left(t_{k}\right)= & \frac{1}{\left|N_{i}\right|+m\left|N_{i}^{\prime }\right|+1}\left\{\sum_{j\in N_{i}}\eta_{ij}\left(t_{k}\right)\alpha_{i}\left(t_{k}\right)\right.\\ \; & \left.+\sum_{l\in N_{i}^{\prime }}{\tilde{\eta }}_{il}\left(t_{k}\right)\alpha_{i}\left(t_{k}\right)+{\hat{\alpha }}_{i}\left(t_{k}\right)\alpha_{i}\left(t_{k}\right)\right\}\\ s_{i}\left(t_{k}^{+}\right)= & \frac{1}{|N_{i}\left|+m\right|N_{i}^{\prime }|+1}\left\{\sum_{j\in N_{i}}s_{j}\left(t_{k}\right)\right.\\ \; & \left.+\sum_{l\in N_{i}^{\prime }}\sum_{n=1}^{m}\left[s_{l}\left(t_{k-n}\right)-s_{i}\left(t_{k}\right)\right]\right\} \end{array}$$

By further expanding (31) into its complete recurrence formula, we derive

(32) $$\begin{array}{cc} s_{i}\left(t_{k}^{+}\right)= & \frac{1}{|N_{i}\left|+m\right|N_{i}^{\prime }|+1}\left\{s_{i}\left(t_{k}\right)+\sum_{j\in N_{i}}\left[s_{j}\left(t_{k}\right)-s_{i}\left(t_{k}\right)\right]+\right.\\ \; & \left.\sum_{l\in N_{i}^{\prime }}\left[s_{l}\left(t_{k-1}\right)-s_{i}\left(t_{k}\right)\right]+\cdots +\left[s_{l}\left(t_{k-m}\right)-s_{i}\left(t_{k}\right)\right]\right\} \end{array}$$

According to (32), the logical clock skew update satisfies the high-order consensus algorithm proposed in (6). Consequently, after sufficient update iterations, all network nodes will achieve asymptotic consensus in their logical clock skews. Furthermore, compared with the historical difference term recorded within the same period [31], this cross-time difference term, which incorporates both local current states and multi-hop historical states, can effectively capture the time-accumulated deviation of the relative logical clock skew and mitigate the convergence lag.

Second, the logical clock offset compensation of node *i* is estimated by averaging the relative clock offsets of all one-hop and two-hop neighboring nodes; that is,

(33) $${\hat{\beta }}_{i}\left(t_{k}^{+}\right)={\hat{\beta }}_{i}\left(t_{k}\right)+\frac{\sum_{j\in N_{i}}\beta_{ij}\left(t_{k}\right)+\sum_{l\in N_{i}^{\prime }}\beta_{il}\left(t_{k}\right)}{|N_{i}\left|+\right|N_{i}^{\prime }|+1}$$

### 4.3. Implementation of HCTSP

**Figure 3:**
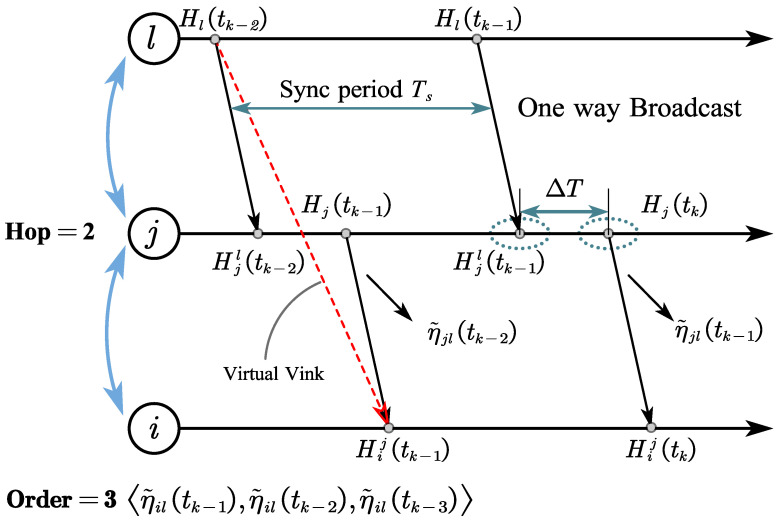
One-way communication model for HCTSP, which uses MAC layer timestamp technology to record the timestamp of the node receiving the RX Time Info.

The corresponding pseudo-code of HSCSP is presented in Algorithm 1. It mainly consists of the following 4 steps:

1. **Asynchronous Periodic Neighbor Broadcasting**: As shown in Figure 3, HCTSP employs a unidirectional broadcast mechanism (see Algorithm 1, lines 3–5). Due to different clock skews, each node autonomously determines its broadcast period $T_{s}$ based on its hardware clock, enabling network-wide asynchronous pseudo- periodic broadcasting.
2. **Relative Clock Parameter Estimation**: Upon receiving time information from neighbor node *j*, node *i* calculates both 1-hop and 2-hop neighbors relative clock parameters $\left\{\eta_{ij}\left(t_{k}\right),\beta_{ij}\left(t_{k}\right)\right\}$, $\left\{{\tilde{\eta }}_{il}\left(t_{k}\right),\beta_{il}\left(t_{k}\right)\right\}$ by processing the received timestamps $\langle H_{j}\left(t_{k}\right),L_{j}\left(t_{k}\right),{\hat{\alpha }}_{j}\left(t_{k}\right)\rangle$ and shared information $\langle {\tilde{\eta }}_{jl}\left(t_{k-1}\right),{\bar{\beta }}_{jl}\left(t_{k-1}\right)\rangle$.
3. **Local Clock Compensation**: As shown in Figure 2, each node calculates its current compensation parameters $\{{\hat{\alpha }}_{i}\left(t_{k}\right)$, ${\hat{\beta }}_{i}\left(t_{k}\right)\}$ by integrating the relative clock parameters output by the high-order state estimator and the previous compensation values. These parameters are then applied to adjust the logical clock $L_{i}\left(t_{k}\right)$.
4. **High-order Information Storage**: Node *i* maintains clock state information by storing $\eta_{ij}\left(t_{k}\right)$ of all neighbors between consecutive broadcast rounds, and then calculates ${\tilde{\eta }}_{ij}\left(t_{k-1}\right)$ for the construction of next virtual links.

Next, we present a detailed description of the HCTSP implementation, focusing on its two core operational aspects: information broadcasting and parameter updating.

Unlike traditional multi-hop relay forwarding, HCTSP adopts a single-hop communication model to reduce the complexity and communication overhead associated with high-frequency packet forwarding. Each broadcast packet contains the sending timestamp, including the hardware and logical clocks, and the calculated relative clock parameters for both one-hop and multi-hop neighboring nodes collected during the broadcast interval. Notably, prior work, i.e., VTSP [25], requires embedding all historically stored relative clock skews of neighboring nodes in broadcast packets to construct virtual links with multi-hop nodes, thereby introducing additional communication overhead.
**Algorithm 1** Pseudo-code algorithm of HCTSP (taking 2-hop communication and 3-order algorithm as an example, i.e., *H* = 2, *M* = 3) where $j\in N_{i}$, $l\in N_{j}$.  1:**Initialization:**  2:   Set ${\hat{\alpha }}_{j}\left(t_{0}\right)=1$, ${\hat{\beta }}_{j}\left(t_{0}\right)=0$, $\eta_{ij}\left(t_{0}\right)=1$, ${\tilde{\eta }}_{jl}\left(t_{0}\right)\to 0$.  3:   Any node *j* broadcasts timing message periodically with period $T_{s}$.  4:**Upon node** ***j*** **triggers the broadcast task**  5:   Broadcast $\langle H_{j}\left(t_{k}\right),L_{j}\left(t_{k}\right),{\hat{\alpha }}_{j}\left(t_{k}\right),{\tilde{\eta }}_{jl}\left(t_{k-1}\right),{\bar{\beta }}_{jl}\left(t_{k-1}\right)\rangle$ to all neighbors.  6:**Upon node** ***i*** **receives** ***j*** **’s message**  7:   Store ${\hat{\alpha }}_{j}\left(t_{k}\right)$, ${\tilde{\eta }}_{jl}\left(t_{k-1}\right)$, ${\bar{\beta }}_{jl}\left(t_{k-1}\right)$ and $|N_{j}|$;  8:   Record reception timestamp $H_{j}\left(t_{k}\right)$;  9:   Calculate $\eta_{ij}\left(t_{k}\right)$, and  update $\left\{\eta_{ij}\left(t_{k}\right)\right\}\leftarrow \eta_{ij}\left(t_{k}\right)$;10:  Calculate $\beta_{ij}\left(t_{k}\right)$, and  update $\left\{\beta_{ij}\left(t_{k}\right)\right\}\leftarrow \beta_{ij}\left(t_{k}\right)$.11:**Calculate shared parameters for real-time**12:   ${\tilde{\eta }}_{ij}\left(t_{k-1}\right)=\sum_{j\in N_{i}}\left\{\eta_{ij}\left(t_{k}\right)\right\}$;13:   ${\bar{\beta }}_{ij}\left(t_{k-1}\right)=\frac{\sum_{j\in N_{i}}\left\{\beta_{ij}\left(t_{k}\right)\right\}}{\left|N_{i}\right|+1}$.14:**if $t_{k}>M$ then**15:   Use FIFO rule to perform sliding storage for ${\left\{\eta_{jl}\left(t_{k-1}\right)\right\}}_{l\in {N_{i}}^{\prime }}$;16:   Update ${\left\{\eta_{jl}\left(t_{k-1}\right)\right\}}_{l\in N_{i}^{\prime }}\leftarrow {\tilde{\eta }}_{jl}\left(t_{k}\right)$, and calculate ${\tilde{\eta }}_{jl}\left(t_{k-1}\right)=\sum {\left\{\eta_{jl}\left(t_{k-1}\right)\right\}}_{l\in N_{i}^{\prime }}$;17:   Calculate ${\tilde{\eta }}_{il}\left(t_{k}\right)=\alpha_{ij}\left(t_{k}\right){\tilde{\eta }}_{jl}\left(t_{k-1}\right)$, and update ${\left\{{\tilde{\eta }}_{il}\left(t_{k}\right)\right\}}_{l\in N_{i}^{\prime }}\leftarrow {\tilde{\eta }}_{il}\left(t_{k}\right)$;18:   Calculate $\beta_{il}\left(t_{k}\right)=\beta_{ij}\left(t_{k}\right)+{\bar{\beta }}_{jl}\left(t_{k-1}\right)$, and update ${\left\{\beta_{il}\left(t_{k}\right)\right\}}_{l\in N_{i}^{\prime }}\leftarrow \beta_{il}\left(t_{k}\right)$.19:**end if**20:**Upon node** ***i*** **triggers the update time**21:   Calculate ${\hat{\alpha }}_{i}\left(t_{k}\right)$ and ${\hat{\beta }}_{i}\left(t_{k}\right)$;22:   Update $L_{i}\left(t_{k}\right)$;23:   Update $\langle H_{j}\left(t_{k-1}\right),H_{i}\left(t_{k-1}\right)\rangle \leftarrow \langle H_{j}\left(t_{k}\right),H_{i}\left(t_{k}\right)\rangle$.24:**End**

Although VTSP alleviates the overhead problem through redundant data filtering and passive listening by edge nodes, direct information aggregation may be unreasonable for large-scale dense networks. To avoid this shortcoming, HCTSP introduces an accumulator-based redundant information optimization scheme (see Algorithm 1, lines 11–13). Upon receiving the timing information, node *i* calculates the relative clock skew $\eta_{ij}\left(t_{k}\right)$ and stores it in local memory ${\left\{\eta_{ij}\left(t_{k}\right)\right\}}_{j\in N_{i}}$. Moreover, the local memory of node *i* performs real-time accumulation of the calculated relative clock skew values of all neighboring nodes *j*. Meanwhile, the accumulator result ${\tilde{\eta }}_{ij(t_{k-1})}$ is output when the broadcasting task of node *i* is triggered and is used to construct multi-hop virtual links for the non-neighboring nodes of node *i*. Taking Figure 1 as a representative case, by (28), the virtual link is constructed as

(34) $$\begin{array}{cc} \eta_{il}\left(t_{k}\right)= & \alpha_{ij_{1}}\left(t_{k}\right)\eta_{j_{1}l_{1}}\left(t_{k}\right)+\alpha_{ij_{1}}\left(t_{k}\right)\eta_{j_{1}l_{2}}\left(t_{k}\right)\\ \; & +\alpha_{ij_{2}}\left(t_{k}\right)\eta_{j_{2}l_{2}}\left(t_{k}\right)+\alpha_{ij_{2}}\left(t_{k}\right)\eta_{j_{2}l_{3}}\left(t_{k}\right) \end{array}$$

Although threshold processing [25] can filter out duplicate information and ensure that only the most concise virtual link exists between node *i* and non-adjacent node *l*, it may inadvertently discard potentially valid virtual links, thereby compromising convergence speed. In contrast, HCTSP circumvents this issue through real-time local memory computation; that is,

(35) $$\begin{array}{cc} \eta_{il}\left(t_{k}\right) & =\alpha_{ij_{1}}\left(t_{k}\right)\left(\eta_{j_{1}l_{1}}\left(t_{k}\right)+\eta_{j_{1}l_{2}}\left(t_{k}\right)\right)\\ \; & \;\;+\alpha_{ij_{2}}\left(t_{k}\right)\left(\eta_{j_{2}l_{2}}\left(t_{k}\right)+\eta_{j_{2}l_{3}}\left(t_{k}\right)\right)\\ \; & =\alpha_{ij_{1}}\left(t_{k}\right)\sum_{l\in N_{j_{1}}}\eta_{j_{1}l}\left(t_{k}\right)+\alpha_{ij_{2}}\left(t_{k}\right)\sum_{l\in N_{j_{2}}}\eta_{j_{2}l}\left(t_{k}\right) \end{array}$$

Furthermore, let

(36) $${\tilde{\eta }}_{jl}\left(t_{k}\right)=\sum_{l\in N_{j}}\eta_{jl}\left(t_{k}\right)$$

So ${\tilde{\eta }}_{il}\left(t_{k}\right)$ ultimately derives the following form; that is

(37) $$\eta_{il}\left(t_{k}\right)=\sum_{j\in N_{i}}\alpha_{ij}\left(t_{k}\right){\tilde{\eta }}_{jl}\left(t_{k}\right)$$

Through this optimization, sending node *j* compresses the additional synchronization information into one data unit instead of the $|N_{j}|$ neighboring data units required by the traditional full-message embedding scheme, thereby effectively reducing the packet size.

Subsequently, we focus on the parameter updating process to refine HCTSP. The update phase encompasses four key operations: (a) local timestamp recording upon message reception (lines 6–8), (b) calculation of relative clock parameters between adjacent nodes (lines 9–10), (c) calculation of relative clock parameters between the local node and its multi-hop nodes (lines 14–18), and (d) calculation of logical clock compensation values followed by logical clock synchronization (lines 20–22).

The critical step of the update phase is (c). Upon receiving timestamps and shared multi-hop information, the local node first derives the one-hop logical skew compensation $\eta *ij(t_{k})$ and offset $\beta *ij(t_{k})$ based on (26). Then, a high-order consensus algorithm is employed to estimate the multi-hop logical skew compensation $\tilde{\eta }*il\left(t_{k}\right)$ using both current-round and stored historical multi-hop information. Concurrently, a virtual link with offset $\beta *il=\beta_{ij}\left(t_{k}\right)+\bar{\beta }*jl\left(t*k-1\right)$ is constructed using the shared parameter $\bar{\beta }*jl\left(t*k-1\right)$ forwarded by neighboring nodes, while the local relative clock parameter table $\langle \tilde{\eta }*il\left(t_{k}\right)*i\in N_{i}^{\prime },\beta_{il}\left(t_{k}\right)*i\in N*i^{\prime }\rangle$ is refreshed. Finally, at the preset update time, each node calculates the two crucial compensation parameters ${\hat{\alpha }}_{i}\left(t_{k}\right)$ and $\hat{\beta_{i}}\left(t_{k}\right)$ for logical clock adjustment based on (30) and (33), thereby achieving logical clock synchronization.

(38) $$L_{i}\left(t_{k}\right)={\hat{\alpha }}_{i}\left(t_{k}\right)H_{i}\left(t_{k}\right)+{\hat{\beta }}_{i}\left(t_{k}\right)$$

### 4.4. Performance Comparison Analysis

#### 4.4.1. Convergence Speed

Building on similar virtual-link frameworks [21,23,25], HCTSP further optimizes convergence speed by integrating high-order consensus theory. Specifically, it introduces more historical information from multi-hop nodes to accelerate the convergence speed of the average consensus algorithm. The detailed theoretical proof has been provided in Section 3.2.

#### 4.4.2. Communication Overhead

Considering a two-hop virtual-link topology where each node has an average degree of *d* as a case study, in MACTS, each node performs an immediate relay-forwarding communication mechanism to establish virtual links. For each node, MACTS requires $d^{2}$ additional forwarding communication operations to establish contact with two-hop neighbors. In contrast, VTSP, PACTS, and HCTSP employ a single-hop communication model to save significant communication overhead. However, VTSP simply embeds the received timing messages of *d* neighboring nodes into the current synchronization packet. The packet size increases linearly with the number of neighbors, inevitably leading to increased communication cost and scalability limitations in dense IWSNs. For PACTS and HCTSP, these two protocols require only one aggregated estimation of all neighboring clock states rather than individual neighboring information, greatly reducing the redundant packet overhead in VTSP. Although PACTS and HCTSP adopt similar techniques to reduce packet overhead, the previous analysis has shown that HCTSP can converge faster, resulting in lower communication overhead at the same synchronization accuracy.

#### 4.4.3. Synchronization Accuracy

Communication delay is a critical factor affecting the performance of CTSPs. In virtual-link-based synchronization protocols, multi-hop information is inevitably affected by delay uncertainty, which may degrade synchronization stability and accuracy. Unlike conventional methods that directly rely on current-round multi-hop estimates, HCTSP incorporates finite-order historical multi-hop information into the skew estimation process. The cross-time difference term $s_{l}\left(t_{k-m}\right)-s_{i}\left(t_{k}\right)$ reduces the dependence on a single delayed estimate and provides a smoothing effect against instantaneous delay jitter.

The synchronization error of HCTSP mainly consists of two components: the propagation error introduced by virtual multi-hop links and the historical-delay error associated with the finite-memory mechanism. The former generally increases with hop count, whereas the latter is constrained by the memory order *m* and update interval ΔT. Therefore, incorporating historical information does not imply unconditional suppression of delay-induced errors. Its effectiveness depends on the balance between delay accumulation and historical-delay compensation [36].

In this work, the delay jitter considered in Section 5.3 remains relatively small compared with the synchronization period. Under such conditions, the finite-memory mechanism can effectively smooth transient delay fluctuations, thereby reducing sensitivity to instantaneous jitter and improving synchronization accuracy. Nevertheless, when the hop count, memory order, or delay jitter becomes sufficiently large, the accumulated delay error may outweigh the benefits provided by historical information, leading to degraded synchronization performance.

Based on the above theoretical analysis and design, Table 1 presents a performance comparison between the proposed method and existing GTSP-based and ATS-based algorithms to intuitively demonstrate the superiority of the proposed high-order virtual-link mechanism. Detailed simulation results and corresponding analyses are provided in Section 5.

## 5. Simulation Results

This section evaluates the performance of HCTSP through MATLAB R2018b simulations by comparing it with the traditional single-hop protocols GTSP [4] and ATS [5], as well as the two-hop protocols VTSP [25] and PACTS [23], under a typical two-hop virtual communication scenario. The initial logical clock parameters are set as ${\hat{\alpha }}_{i}\left(0\right)=1$, $\hat{\beta }*i\left(0\right)=0$, and α∗ij(0)=1, while the broadcast period $T_{s}$ of each node is set to 1 s. Given that sensor nodes typically exhibit a clock drift ranging from 10 to 100 ppm [37], we initialize the hardware clock skew as $\alpha_{i}\left(0\right)\in \left[0.9999,1.0001\right]$ and the clock offset as $\beta_{i}\left(0\right)\in \left[0,0.0002\right]$ s [7]. The crystal oscillator frequency is set to 32.768 kHz. Moreover, the number of virtual communication hops *H* is set to 2, and the memory order *m* in HCTSP is configured as 3. Note that ε is introduced in the theoretical analysis to establish the convergence condition. Since HCTSP follows the normalized GTSP framework, the practical parameter study mainly focuses on the memory order *m*.

Let the global logical clock and skew differences, i.e., $\Delta_{g,L}\left[t_{k}\right]=\max\left\{\left|L_{j}\left[t_{k}\right]-L_{i}\left[t_{k}\right]\right|\right\}$ and $\Delta_{g,S}\left[t_{k}\right]=\max\left\{\left|{\hat{\alpha }}_{j}\left[t_{k}\right]\alpha_{j}\left[t_{k}\right]-{\hat{\alpha }}_{i}\left[t_{k}\right]\alpha_{i}\left[t_{k}\right]\right|\right\}$, ∀{i,j}∈V, denote the synchronization state. As the primary performance metric in this study, convergence speed is evaluated by measuring the time required for each protocol to reach a predefined synchronization error threshold. According to the industrial automation network standards ISA100.11a and WirelessHART [38], the typical synchronization accuracy requirement for wireless sensors in industrial production scenarios ranges from ±90 to 100 µs. Therefore, we set the synchronization error threshold to 2 µs, which is a significantly stricter criterion than the industrial standard. When the system synchronization error satisfies Δ∗g,L<2 µs, the network is considered to have achieved acceptable consensus convergence.

As the proposed HCTSP shares the same fundamental update principle as GTSP, the comparative analysis mainly focuses on GTSP-related protocols. Moreover, to demonstrate the general applicability of high-order consensus theory, we also investigate its application to ATS.

### 5.1. Convergence Performance Comparison

**Figure 4:**
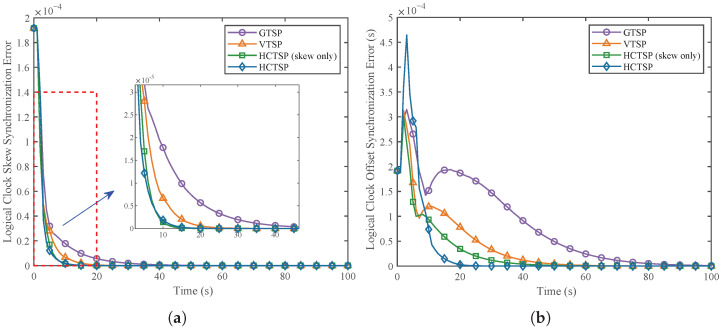
Comparison of global synchronization error on grid topology. (**a**) Logical clock skew sync-error; (**b**) Logical clock offset sync-error.

Figure 4 demonstrates the convergence characteristics of the logical clock under different protocols in a 6 × 6 grid topology. It should be noted that the vertical axis in Figure 4b represents the absolute skew value, which is dimensionless and therefore has no unit. Obviously, the proposed HCTSP exhibits faster convergence than GTSP and VTSP. To systematically evaluate the contribution of the joint skew-offset dual-channel virtual-link mechanism in HCTSP, we conducted comparative experiments involving a skew-only virtual link and a joint skew-offset virtual link. The performance improvement achieved by the joint skew-offset mechanism can be clearly observed from the results.

It should be noted that all protocols exhibit an initial surge in offset error during the early stage of synchronization. The reason for this phenomenon is that the initial logical skew error amplifies the logical offset error. Meanwhile, since the logical offset update incorporates multi-hop historical synchronization data, which inherently introduces larger initial errors, this phenomenon is particularly pronounced in HCTSP (skew-only). However, as the logical skew gradually converges, the logical offset becomes the dominant factor in the iterative process, and the advantages of the joint skew-offset compensation mechanism become apparent.

**Figure 5:**
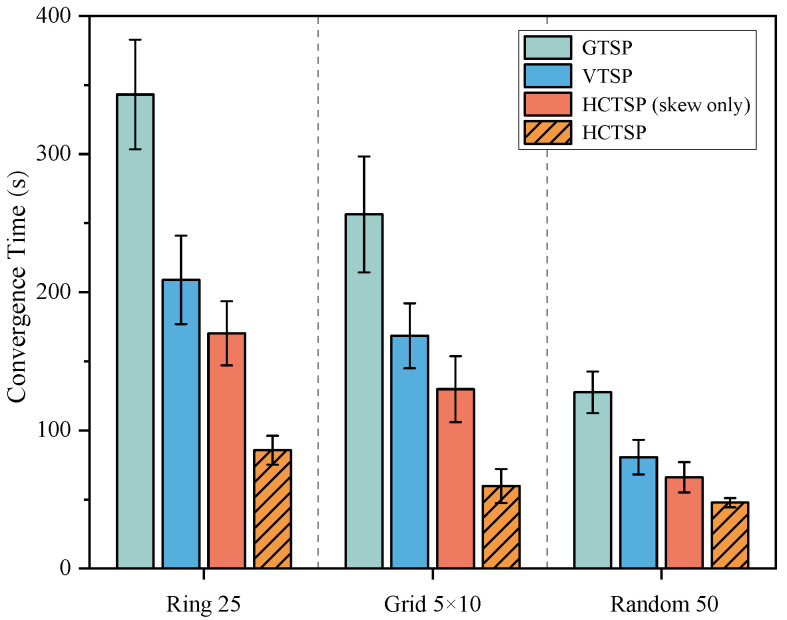
Statistical graph of convergence speed in different topologies.

Next, we performed a systematic comparison of the convergence speed of different GTSP-based protocols under various network topologies. The experimental data presented in Figure 5 and Table 2 represent the statistical results obtained from 20 independent runs under ring, 5 × 10 grid, and random topologies. For the random topology, 50 nodes were randomly deployed within a 150 × 150 area with a communication radius of $\sqrt{30}$. It is well known that the convergence time of CTSPs is strongly related to the algebraic connectivity of the network topology. Therefore, in the sparse ring-topology environment, the convergence speed of all protocols is the slowest. In contrast, HCTSP can still achieve relatively fast convergence even in a ring topology.

Furthermore, Figure 5 reveals a notable difference in convergence speed between the grid and random topologies despite their identical network scale (50 nodes). This difference mainly stems from network complexity, i.e., network density, which can be calculated as ψ=2|E|/(N(N−1)) [39]. Therefore, network complexity is determined by both the number of nodes and the number of communication links. In other words, the denser the topology, the faster the convergence speed. To systematically investigate this relationship, we further conducted a comprehensive experimental study under randomized topological conditions. The random network topology configurations are listed in Table 3.

**Figure 6:**
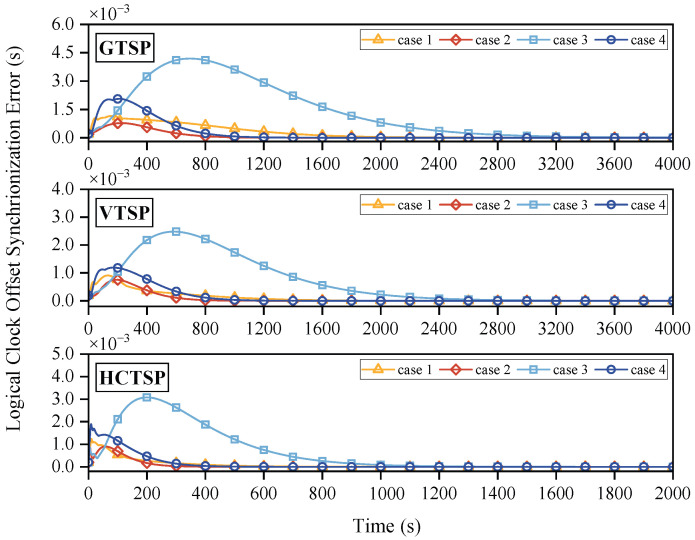
Convergence speed of GTSP—VTSP—HCTSP in Cases 1–4.

It can be seen from the comparison results of various consensus protocols in Figure 6 that network complexity is a key factor limiting the convergence speed of average consensus protocols. Obviously, regardless of the protocol, Case 2, which has the highest network complexity, achieves the fastest convergence. Meanwhile, for a fixed network size, a larger network area may result in a larger network diameter *D*. This parameter directly affects the algebraic connectivity $\lambda_{2}$ of the network, i.e., ${\left(1/D\right)}^{2}\leq \lambda_{2}\leq \left(2d/D\right)$. Therefore, the larger the network diameter *D*, the slower the network convergence speed.

Specifically, although Cases 1 and 3 maintain the same number of nodes (N=100), the larger sensing area in Case 3 results in a greater maximum hop count (i.e., network diameter) and fewer communication links than in Case 1, thereby leading to slower convergence speed.

The theoretical results in Section 3.2 indicate that a higher order of HCTSP leads to faster convergence. However, in topologies with high network density, excessively high orders may introduce more historical information, thereby increasing delay jitter and network congestion among nodes and ultimately affecting convergence performance. To determine the optimal order of HCTSP, we further conducted a large number of statistical experiments. The simulation parameters are consistent with those used in Figure 5.

**Figure 7:**
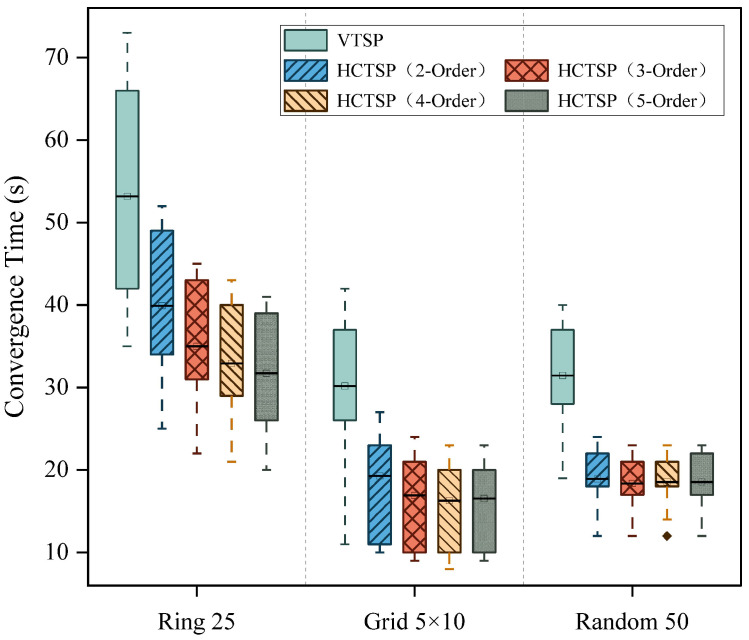
Comparison of orders under the same number of hops.

The simulation results in Figure 7 demonstrate a clear positive correlation between the consensus order and convergence speed in sparse networks such as ring topologies. Nevertheless, the convergence speed does not continue to improve indefinitely as the order increases in grid and random dense topologies. When the order reaches 3 or higher, the convergence speed no longer improves and may even exhibit a slight decrease, particularly in random topologies. Therefore, it is recommended to use HCTSP with an optimal order of 3.

### 5.2. Communication Overhead Comparison

The power consumption of nodes in IWSNs mainly originates from the transceivers of wireless communication modules. Therefore, this section analyzes the communication overhead by comparing the number of packet exchanges.

In GTSP, VTSP, and HCTSP, each node with $d_{i}$ neighbors exchanges $1+d_{i}$ packets within a synchronization cycle, including one synchronization message broadcast and $d_{i}$ receptions of timestamp information from neighboring nodes. Hence, the total number of packet exchanges required to complete one synchronization round across the entire network is $N_{d}=\sum_{i=1}^{n}\left(1+d_{i}\right)$. Although these protocols have the same number of message exchanges in a single iteration, their convergence times differ. Here, we define the communication overhead ratio *R* as the performance metric, where $R=N_{P_{E}}/N_{T_{M}}$. $N_{P_{E}}$ represents the number of packet exchanges required to reach the convergence threshold, and $N_{T_{M}}$ denotes the total number of packet exchanges within the simulation time $T_{M}$. In this section, $T_{M}$ is set to 100 s. Clearly, if R>1, it indicates that the protocol fails to converge within the predefined simulation time. Conversely, a lower value of *R* indicates lower communication overhead.

**Figure 8:**
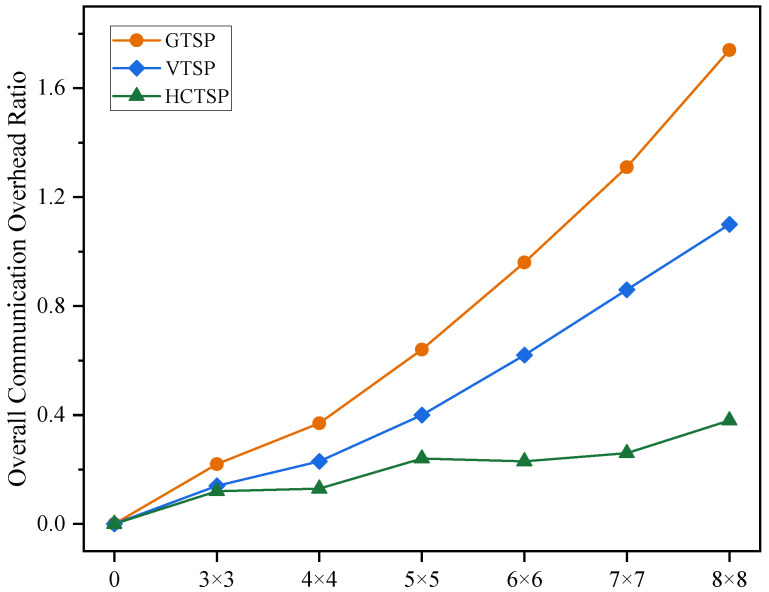
Total network overhead ratio of GTSP-based protocols.

Figure 8 shows the communication overhead ratios of each protocol under different grid-topology sizes when achieving the predefined convergence threshold. Clearly, the proposed HCTSP achieves lower communication overhead. Meanwhile, as the network size increases, the communication overhead also exhibits an increasing trend.

**Figure 9:**
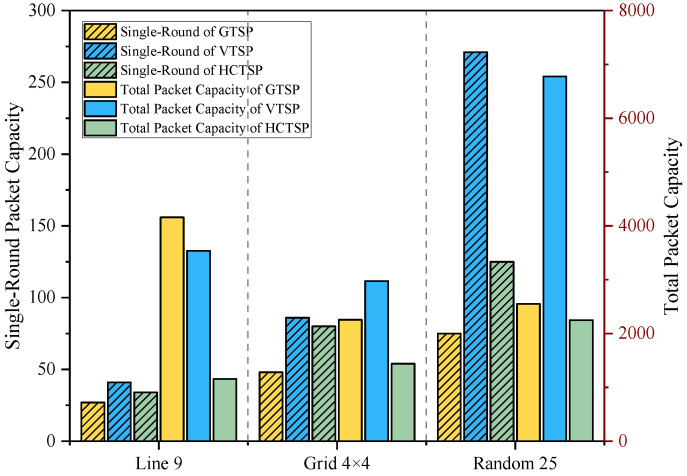
Comparison of packet overhead in GTSP-based protocols.

As shown in Figure 9, this advantage becomes particularly evident in random topologies with dense node distributions. The packet size of HCTSP, optimized through the proposed redundant-information compression scheme, is reduced to approximately one-half of that of VTSP, while the final total packet volume is approximately one-third of VTSP. In addition, due to the slower convergence speed of GTSP, comprehensive evaluation shows that HCTSP also requires significantly lower total packet volume across the entire network and demonstrates superior performance in controlling communication overhead.

### 5.3. Synchronization Accuracy Against Communication Delay

Although MAC-layer timestamp technology can effectively mitigate most communication delays and enable precise timestamping, unavoidable delay jitter uncertainties still exist during multi-hop packet transmission. Therefore, the experiment employs a Gaussian distribution with a mean of 2.5 × 10^−6^ s and a variance of 6 × 10^−13^ s^2^ to simulate delay jitter. This indicates that the non-negative delay varies randomly within the approximate range of [0, 5] µs and has a confidence interval of 99.97%.

**Figure 10:**
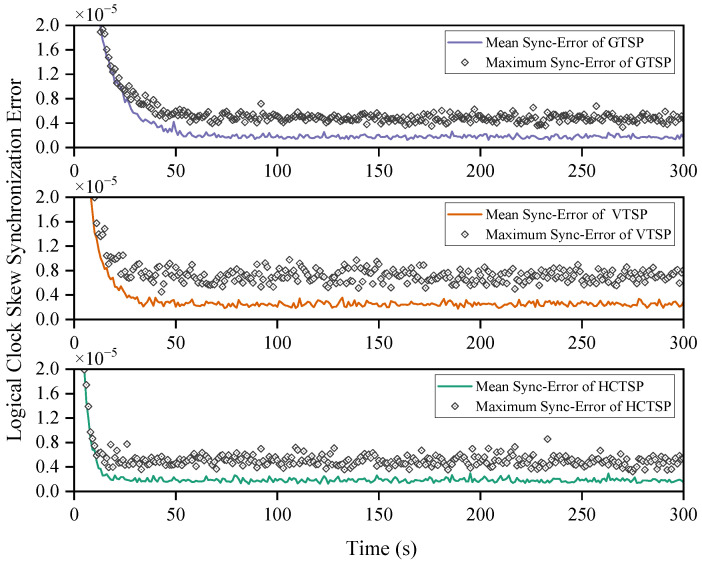
Logical clock skew synchronization errors under Gaussian delay jitter.

In the delayed-network scenario with an 8 × 8 grid topology, Figure 10 shows the statistical synchronization-error results obtained from 20 independent runs. It can be seen that HCTSP still converges the fastest among all protocols under communication-delay conditions. However, the cumulative nature of delays and the lag in virtual-link shared information exacerbate the impact of delays on the estimation accuracy of synchronization parameters. Specifically, in single-hop protocols, delays affect only individual timestamp measurements, whereas in multi-hop networks, both transmission and processing delays accumulate at each hop. Additionally, in an *m*-hop virtual network, the shared information used by the local node at time $t_{k}$ can be traced back to time $t_{k-m}$, thereby causing inaccuracies in the error estimation of both the local node and its neighboring nodes.

As illustrated in Figure 10, these factors result in lower synchronization accuracy for VTSP than for GTSP. Quantitative analysis reveals that while GTSP maintains a mean synchronization error of 2 and a maximum synchronization error of 5, the mean and maximum errors of VTSP can reach approximately 3.5 and 10, respectively. In contrast, HCTSP utilizes historical information from previous rounds of neighboring nodes in local memory, effectively mitigating the impact of cumulative delay on local parameter estimation in multi-hop scenarios and thereby limiting the mean and maximum synchronization errors to approximately 2.5 and 6.

**Figure 11:**
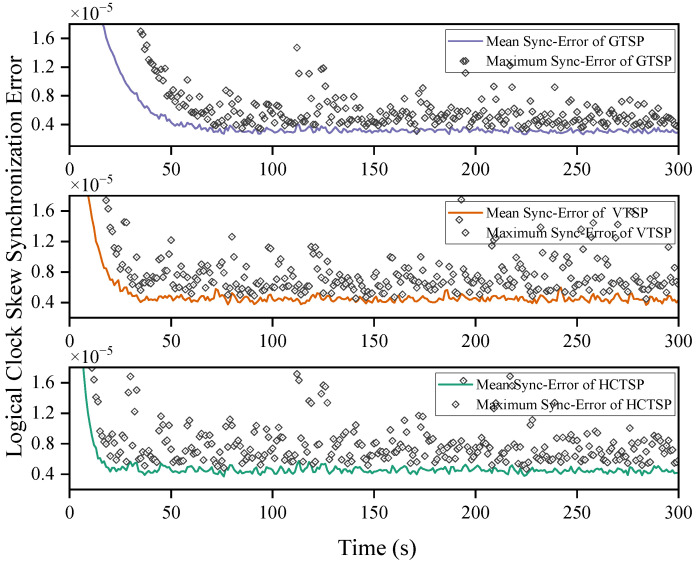
Logical clock skew synchronization errors under Heavy-tailed delay jitter.

**Figure 12:**
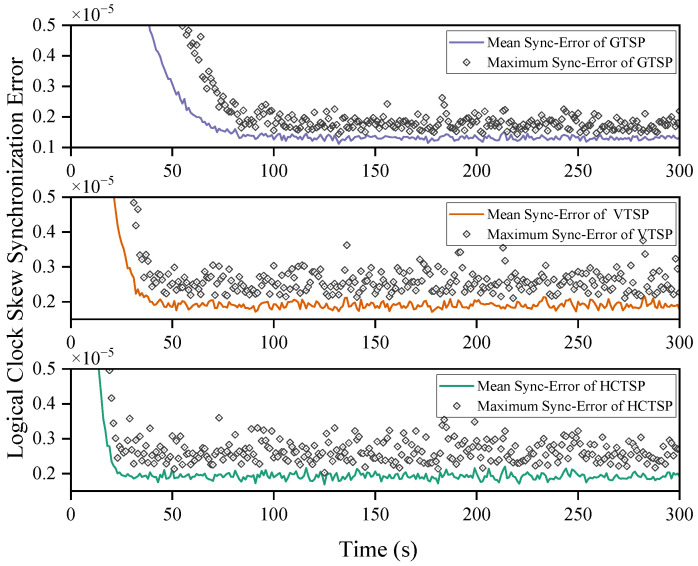
Logical clock skew synchronization errors under Exponential delay jitter.

To further evaluate the robustness of the proposed synchronization scheme under non-Gaussian communication delays, two additional delay models were considered, namely a heavy-tailed delay model and an exponential delay model. The heavy-tailed delay was generated using a Student’s t-distribution with a degree of freedom of ν=3, while the exponential delay was modeled as $d=d_{0}+\mathrm{exprnd}\left(\beta \right)$, where $d_{0}=2.0\times 10^{-6}\;s$ and $\beta =0.5\times 10^{-6}\;s$. The corresponding results are shown in Figure 11 and Figure 12. Under these asymmetric delay conditions, HCTSP still exhibits the fastest convergence among the three protocols owing to the utilization of historical synchronization information. However, the bursty characteristics of non-Gaussian delays weaken the smoothing effect provided by the historical-memory mechanism. Consequently, although HCTSP still achieves slightly better synchronization accuracy than VTSP, the improvement becomes less pronounced than that observed under Gaussian delay conditions.

**Figure 13:**
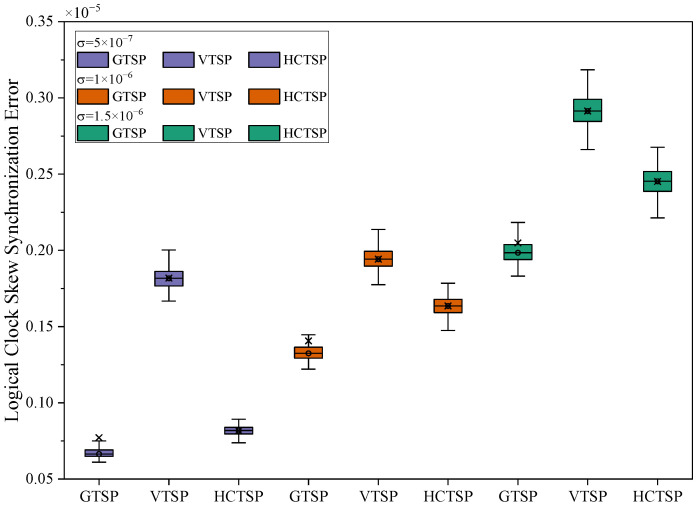
Synchronization errors with different standard deviations with a 68% confidence interval.

**Figure 14:**
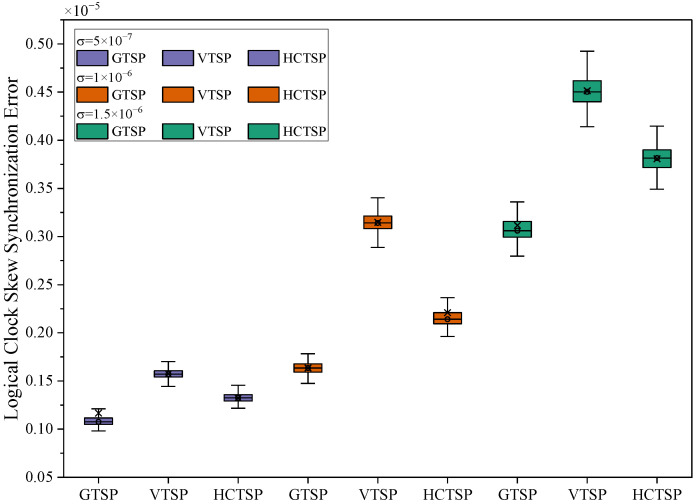
Synchronization errors with different standard deviations with a 95% confidence interval.

Furthermore, to evaluate the statistical performance under different levels of Gaussian delay jitter, three standard deviations were considered, namely $\sigma =5\times 10^{-7}\;s$, $\sigma =1\times 10^{-6}\;s$, and $\sigma =1.5\times 10^{-6}\;s$. For each setting, 20 independent simulation runs were performed with randomly generated initial clock offsets and communication delays while maintaining the same network topology and protocol parameters. To eliminate the influence of the transient synchronization process, the first 50 iterations were excluded, and only the mean synchronization error during the steady-state phase was considered. Box plots were then constructed under the 68% and 95% confidence levels, as shown in Figure 13 and Figure 14. As the standard deviation of the delay increases, the synchronization errors of all three protocols increase accordingly. GTSP maintains the lowest median synchronization error, while HCTSP achieves slightly better synchronization accuracy than VTSP while maintaining a faster convergence rate.

### 5.4. Universality Analysis

The ATS protocol [5,21,23] represents another branch of average-consensus-based CTSPs. Recent advancements have led to the development of PACTS [23], which also incorporates a virtual-link mechanism to enhance convergence speed. Unlike the synchronous update strategy of GTSP, the asynchronous instantaneous update mechanism of ATS provides a higher update frequency, resulting in faster convergence than GTSP. Therefore, to verify the universality of the proposed method, we further extend the experimental evaluation to include a high-order-consensus-based ATS protocol (HATS). The iterative parameters $\rho_{\eta }$, $\rho_{v}$, and $\rho_{o}$ in ATS and PACTS are set to 0.2, 0.5, and 0.5, respectively, while $\rho_{v}$ in HATS is set to 0.25. The order of HATS is configured as 2. The remaining simulation parameters are consistent with those of HCTSP.

**Figure 15:**
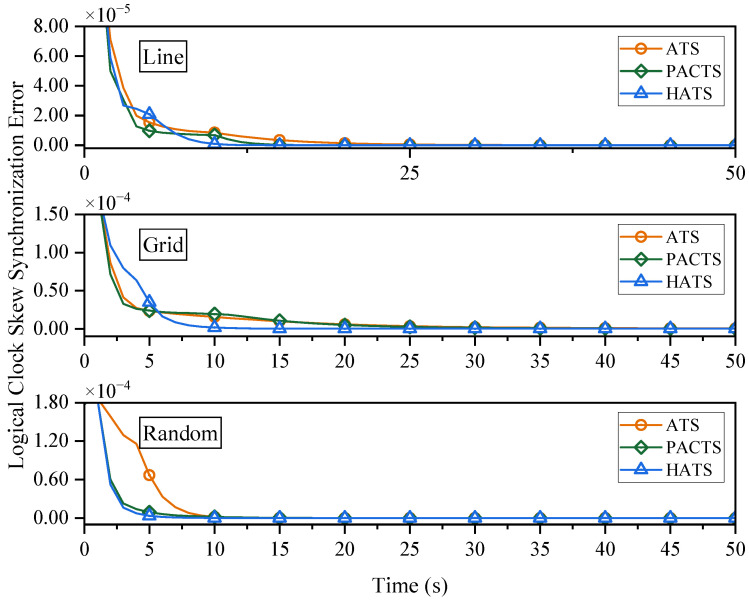
Logical clock skew sync-error in ATS-based protocols.

**Figure 16:**
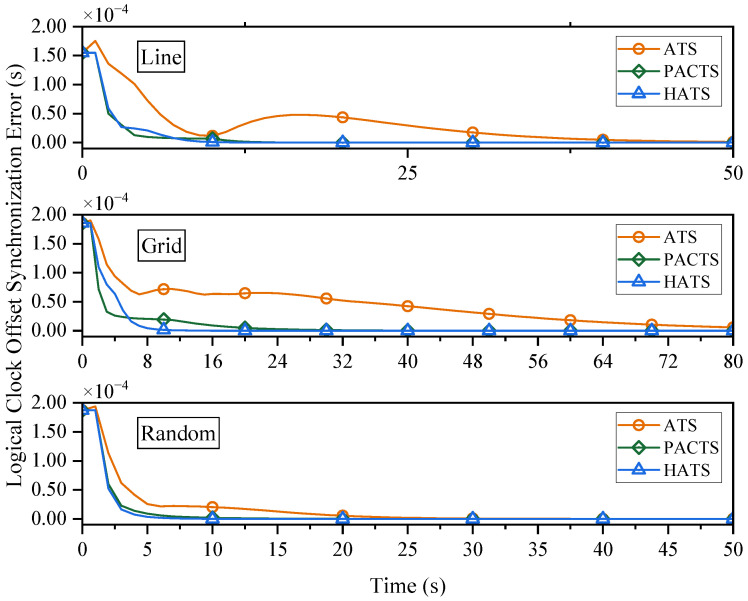
Logical clock offset sync-error in ATS-based protocols.

We first analyze the convergence performance of HATS in comparison with ATS and PACTS under various topologies, including a line topology with 30 nodes, a 5 × 10 grid topology, and random topologies consisting of 60 nodes randomly deployed within a 150 × 150 area with a communication radius of $\sqrt{30}$. The simulation results shown in Figure 15 and Figure 16 demonstrate that the proposed high-order-consensus-based virtual-link mechanism can still maintain faster convergence in ATS.

**Figure 17:**
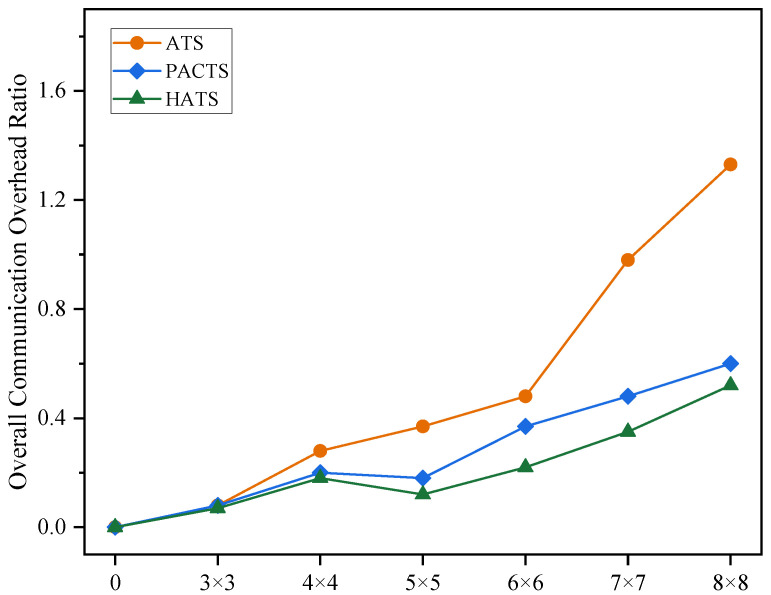
Communication overhead ratio of ATS-based protocols.

Figure 17 further compares the communication-overhead ratio of each protocol when achieving the predefined convergence threshold under different topology scales. Clearly, compared with the single-hop ATS protocol and the multi-hop PACTS protocol, the proposed high-order-consensus-based virtual-link ATS protocol consistently achieves the desired synchronization error bound with the fewest packet exchanges.

**Figure 18:**
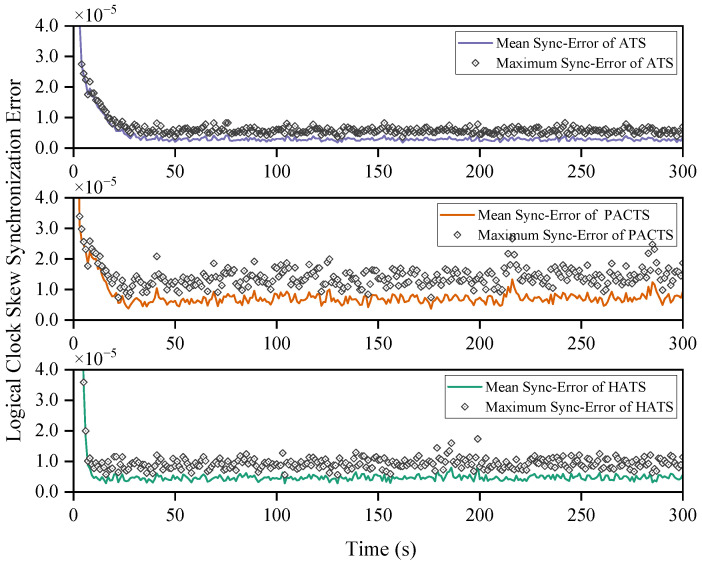
Logical clock skew synchronization errors under delay jitter.

Finally, we conducted the experiment shown in Figure 18 using the same delay-network model described in Section 5.3. It is well known that communication delay mainly affects the estimation of the relative clock skew $\alpha_{ij}$ and ultimately influences synchronization accuracy. For multi-hop virtual links (taking the two-hop case as an example), the relative clock skew estimation $\alpha_{il}=\alpha_{ij}\alpha_{jl}$ is subject to compounded errors introduced by two successive delays. Consequently, this leads to reduced robustness and lower synchronization accuracy compared with conventional single-hop ATS. The results presented in Figure 18 further support this analysis.

Although PACTS achieves faster convergence than ATS, quantitative analysis reveals that PACTS maintains a global mean relative-clock-skew error of approximately 9, whereas ATS stabilizes at approximately 5. In contrast, HATS can suppress the cumulative effect of multi-hop delays and ultimately stabilizes at approximately 6.5 while maintaining a faster convergence rate.

## 6. Discussion

### 6.1. Complexity Analysis

The previous analysis shows that the accumulator-based HCTSP effectively reduces the transmission overhead of multi-hop virtual synchronization algorithms. However, for resource-constrained IWSN nodes, algorithm complexity is also a critical consideration. Therefore, this section further compares HCTSP with VTSP and PACTS in terms of computational and storage complexity.

#### 6.1.1. Computational Complexity

The computational complexity mainly arises from one-hop and two-hop relative clock parameter estimation, as well as the updates of logical clock skew and logical clock offset compensation parameters. Let $d_{1}$ and $d_{2}$ denote the numbers of one-hop neighbors and two-hop virtual neighbors, respectively. The computational complexity of both VTSP and PACTS is $O(d_{1}+d_{2})$. In addition, both HCTSP and PACTS introduce aggregation operations to reduce the transmission overhead caused by redundant multi-hop information, thereby incurring additional local computation. Furthermore, since HCTSP exploits two-hop information in both logical clock skew and logical clock offset compensation updates, its practical computational workload per synchronization round is higher than that of VTSP and PACTS. However, these additional operations only increase the constant factor and do not affect the asymptotic complexity. Therefore, the overall computational complexity of HCTSP remains $O(d_{1}+d_{2})$, which is of the same linear order as VTSP and PACTS.

#### 6.1.2. Storage Complexity

Storage complexity is determined by the amount of local memory required to maintain synchronization-related information. For VTSP and PACTS, each node only needs to store one-hop neighbor states and two-hop virtual-neighbor states, resulting in a storage complexity of $O(d_{1}+d_{2})$. In HCTSP, besides maintaining neighbor-state information, each node additionally employs a FIFO buffer to preserve high-order historical data for accumulator-based synchronization. Let *M* denote the FIFO memory order. The FIFO buffer introduces an additional storage overhead of $O(Md_{1})$, yielding an overall storage complexity of $O(d_{1}+d_{2}+Md_{1})$. Since *M* is configured as a small fixed constant in practical implementations, the storage complexity of HCTSP remains linear with respect to network size, and the additional memory requirement introduced by HCTSP is limited. Overall, the moderate increase in computational and storage overhead represents a reasonable trade-off for achieving faster convergence and lower transmission overhead. The computational and storage complexity comparison of VTSP, PACTS, and HCTSP is summarized in Table 4.

### 6.2. Limitations

Although the proposed HCTSP demonstrates promising synchronization performance under the considered scenarios, several issues remain to be further investigated. Future work will focus on the theoretical analysis of high-order virtual links in multi-hop (≥3) scenarios under complex network conditions. In particular, adaptive memory-order selection mechanisms that consider network topology, communication quality, and delay accumulation will be investigated to improve the adaptability of the proposed algorithm in dynamic environments. Moreover, practical factors such as packet loss, node failures, dynamic node participation, burst latency, and temperature-dependent clock drift will be incorporated to further evaluate and enhance the robustness of the synchronization framework. Finally, the performance of the proposed high-order consensus synchronization algorithm will be validated on physical platforms to facilitate its practical deployment.

## 7. Conclusions

This paper has presented a novel high-order consensus time synchronization protocol (HCTSP) that leverages a multi-hop consensus algorithm to address the slow convergence issue in CTSPs. The key contribution of this work is the demonstration that incorporating historical information from nonadjacent nodes can improve clock-skew estimation performance, thereby enhancing convergence efficiency. In addition, the virtual-link construction method based on a single-hop communication model and integrated neighbor-information transmission effectively reduces communication packet overhead. Meanwhile, the distinctive integration of historical and current timestamp information provides enhanced robustness against delay accumulation in multi-hop virtual communication scenarios. Extensive simulation results demonstrate that HCTSP outperforms existing multi-hop consensus protocols in terms of convergence speed, communication overhead, and synchronization accuracy, while also exhibiting good general applicability. 

## Figures and Tables

**Table 1:** Summary of GTSP-based and ATS-based time synchronization protocols.

	GTSP-Based Algorithm			ATS-Based Algorithm		
Indicator	GTSP	VTSP	HCTSP	ATS	PACTS	HATS
Core Mechanism	Single-hop	Virtual Link	High-order Virtual Link	Single-hop	Virtual Link	High-order Virtual Link
Convergence Speed	Slowest	Medium	Fastest	Slowest	Medium	Fastest
Communication Overhead	Low	High	Lowest	High	Low	Lowest
Synchronization Accuracy	High	Medium	Highest	High	Medium	Highest

Note: The performance comparison in Table 1 is conducted among algorithms of the same category.

**Table 2:** Statistical Data of Convergence Time under Different Topologies.

Topology	HCTSP	VTSP	GTSP	Improvement vs. VTSP
Ring	85 s	209 s	344 s	59.33%
Grid	60 s	128 s	256 s	53.13%
Random	48 s	81 s	169 s	40.74%

**Table 3:** Random Topology Cases with A Node Radius of $\sqrt{12}$.

Topology	Area	Size	Edge Number	Network Density
Case 1	90 × 90	100 nodes	217	0.0438
Case 2	90 × 90	120 nodes	339	0.0475
Case 3	100 × 100	100 nodes	205	0.0414
Case 4	100 × 100	120 nodes	306	0.0429

**Table 4:** Computational and storage complexity comparison.

Protocol	Computational Complexity	Storage Complexity
VTSP	$O(d_{1}+d_{2})$	$O(d_{1}+d_{2})$
PACTS	$O(d_{1}+d_{2})$	$O(d_{1}+d_{2})$
HCTSP	$O(d_{1}+d_{2})$	$O(d_{1}+d_{2}+Md_{1})$

## Data Availability

The original contributions presented in this study are included in the article. Further inquiries can be directed to the corresponding author.
